# Mobility On Demand: What About the Weekend?

**DOI:** 10.1177/03611981251346454

**Published:** 2025-08-15

**Authors:** Grace O. Kagho, Milos Balac, Kay W. Axhausen

**Affiliations:** 1 Institute for Transport Planning and Systems (IVT), ETH Zurich, Switzerland; 2 Center for Sustainable Future Mobility (CSFM), ETH Zurich, Switzerland

**Keywords:** Mobility-on-demand, ride-sharing, shared, agent-based, MATSim

## Abstract

Mobility on demand (MoD) services like ride-hailing, ride-sharing, and car-sharing are changing travel behavior by providing increased options and flexibility. These services can be best understood and planned for through the use of detailed computer simulations. However, existing simulations predominantly focus on modeling average working days, characterized by high and predictable travel demand. This approach overlooks the distinct travel patterns observed during weekends. Unlike weekdays, which feature pronounced peak hours, weekend travel is distributed more evenly throughout the day, particularly on Saturdays. This study compares the differences in travel demand patterns between weekends and weekdays and their possible impact on policies drawn from MoD simulations. Using an agent-based simulation framework MATSim, we simulate the introduction of an autonomous mobility on demand (aMoD) service to Zurich, Switzerland, and its environs. We then compare weekday and weekend travel patterns highlighting unique aspects of weekend travel and their implications for MoD service operations. The findings suggest that transport policies should account for the unique characteristics of weekend travel. The results provide insights into modal shifts, showing how more public transport and private vehicle trips could be replaced by MoD services during weekends, especially for long-distance travel. Furthermore, results show that optimal fleet sizes vary between weekdays and weekends owing to differences in demand. While weekends see higher MoD demand, wait times don’t necessarily increase. However, longer detours for pickups may extend travel times. Accounting for weekend travel in simulations helps ensure policy planning supports reliable service while balancing wait times, travel times, occupancy, and operational costs.

There is a growing interest in modeling and simulating various mobility on demand (MoD) services, including ride-hailing, ride-sharing, car-sharing, and even shared autonomous vehicles (SAV). These services are viewed as potential strategies to mitigate transport-related externalities. Agent-based simulations have gained popularity as a robust tool for modeling these MoD services. They provide a detailed, microscopic perspective of complex transport scenarios, thus offering valuable insights into the effects of diverse policy decisions and operational interventions on travel behavior.

MoD services, by nature, offer flexible transport options catering to passenger demand with spatial and temporal flexibility, which can only be properly captured through a microscopic representation of individual travelers and vehicles. Agent-based simulations, thus, can help to understand the impact of such flexible and dynamic behavior on the transport system, and therefore their growing popularity.

Typically, travel demand models, including those for simulating MoD services, are based on average working day scenarios, with the weekend rarely considered. As a result, the model outcomes may not fully capture the overall picture of externalities of travel behaviors, and policy impacts (*1*, *2*). Historically, travel demand models have centered around weekdays, rationalized by the higher travel demand on these days, as well as defined commuting patterns that can be modeled. This is despite the obvious differences between weekdays and weekends. Figure 1 shows the share of trips across the day for the weekday, Saturday, and Sunday based on the 2015 Swiss household travel survey. This figure shows that the peak is most pronounced on the average day, indicating a typical peak hour pattern likely attributable to work and school commutes. The morning peak is less intense on Saturday and Sunday, possibly owing to more flexible schedules or reduced work- and school-related travel.

**Figure 1.**
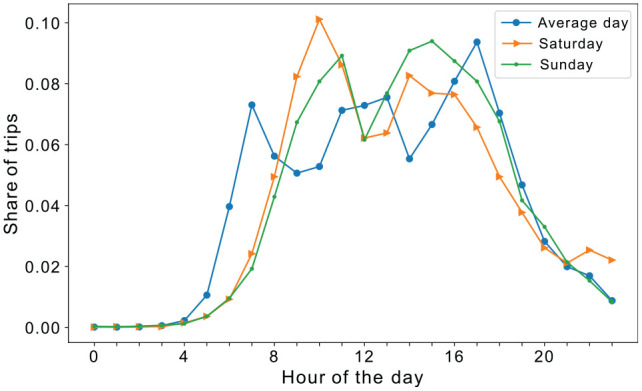
Comparing hourly trip share by day of the week based on the 2015 Swiss Household Travel Survey (Average day represents average of the days of a workweek [weekdays].)

Furthermore, the transportation landscape has changed with the introduction of these flexible modes, which require a better understanding of travel behavior across different days of the week. Many studies have revealed that existing MoD services experience peak demand on weekends, especially on Saturdays. A study analyzing ride-sourcing trips in Austin, Texas, using data from RideAustin showed weekend ride-hailing trips increasing by about 77% on average over weekday trips (*3*). Also, ride-sharing patterns differ between weekdays and weekends as travelers may tend to pool more on the weekends (*3–6*). Consequently, this deviation in travel patterns between weekdays and weekends can substantially affect the planning for these emerging modes and future mobility solutions such as SAVs.

Therefore, including weekend travel data is particularly pertinent for planning and policy decisions related to MoD services, especially in the simulation models used to test these policy scenarios. In this context, our study aims to examine the role of weekend travel in the operational efficiency of MoD services. This is done by first creating a weekend travel demand by adapting an existing travel demand model for an average workday, using weekend-specific travel data to generate activities and activity locations for the model. A simulation of the adoption of MoD services in Zurich, Switzerland, is performed, whereby weekday travel is compared with weekend travel, to provide insights into the unique characteristics of weekend travel and its implications for on-demand service operations and optimization.

The rest of the paper is structured as follows: the second section looks at the relevant background literature. The third section outlines the methodology used. The fourth section discusses the results and their implications in detail, and the fifth section concludes the paper, offering directions for future research in this area.

## Background

Several studies exist that have examined week-long and weekend travel behavior, and they provide an analysis of the differences in travel behavior between weekends and weekdays (*7–12*). These studies argue that special attention needs to be paid to weekend travel to develop a comprehensive travel demand model for evaluating transportation policies to reduce congestion, improve air quality, and enhance well-being. For example, Bhat and Misra (*8*) noted as early as the 1990s that policies focusing on weekday traffic can exacerbate weekend traffic congestion.

The differences in weekend trip patterns are reflected in the activity types, trip length, mode choice, and even duration of trips. Leisure trips account for a higher percentage of weekend trips, and vehicle occupancy is higher because there is more time to participate in household and group activities (*12–15*). For example, the study (*13*) found that weekend mode choice was related to travel party size, while the study (*16*) found in Indonesia that the mode share of fully joint household trips differed between weekdays and weekends. Furthermore, the values of time (VoTs) between weekdays and weekends differs. While some studies report lower VoTs on the weekends than on weekdays (*13*), others suggest higher VoTs on weekends, especially when joint household activities are considered (*15*, *17*). These differences in VoTs can be linked to the various forms of activities and trip chaining patterns, and travel behavior during the weekend compared with the weekday.

These differences in trip patterns also occur between the two weekend days. For example, while the consensus is that weekend trips are generally longer than weekday trips, some studies show that Saturday trips are longer than Sunday trips (*9*), while others find the opposite (*12*). This could depend on the region, so it may be necessary to model Saturday and Sunday independently.

Data from transport network companies (TNC) serve as a rich source for various empirical studies on ride-hailing and ride-sharing. These data are available in the Chicago, New York, Boston, Chengdu, Berlin, and Hamburg regions, and studies based on these data show the differences and similarities between weekday and weekend trip patterns for various travel characteristics (*4–6*, *9*, *18–20*). These studies emphasize the need to model the demand for weekdays and weekends separately. For example, a strong relationship has been found between ride-hailing use and leisure activities (*2*, *4*, *21*), which stands to reason that ride-hailing use is more prevalent on weekends than on weekdays, when people engage in more leisure activities. This can be observed in different regions of the world.

However, the results do not always agree on some points and reveal that the travel patterns depend on the region. The studies by Du et al. (*18*) and Dean and Kockelman (*5*) found that ride-sharing happened more during the week in Chicago (4.5% higher). However, based on TNC data from the pooling-only service MOIA for two German cities, it was found that there were more ride-sharing trips on Saturdays than on any other day of the week (*20*). The study Gehrke et al. (*6*) found that ride-sharing in the Boston area was more prevalent on weekends or in the middle of the day during the week. This suggests that a better analysis of weekend ride-sharing is needed, especially when examining the potential of future mobility services such as SAVs for ride-sharing. In addition, a distinction between Saturday and Sunday is necessary. In Berlin, a study (*22*) using Global Positioning System (GPS trajectories), observed a peak in demand for ride-hailing and ride-sharing trips on Saturdays and lower demand on Sundays. This is similar to other cities such as Madrid, where usage increases on late Friday evenings and on Saturdays (*4*).

Since agent-based simulations are appropriate tools for understanding these MoD services, several MoD simulation studies exist. These simulations model individual agents and their interactions within a transport network, allowing for detailed analysis of travel behavior and the performance of the MoD service. Given that MoD systems are dynamic in adjusting to demand changes in space and time, therefore, agent-based simulations can capture these intricate interactions between supply and demand, whereby each traveler and vehicle are treated as single entities within the transport network, interacting and making decisions in real-time. However, few have addressed the weekend aspect. These are either toy examples (*23*), focused on electric vehicles and their energy demand (*24*, *25*), or choose a weekend day, typically Saturday, considered as the day with the highest demand (*20*, *22*, *26*, *27*). Generally, these studies do not fully explore the differences between weekends and weekdays. As far as the authors are aware, no study examines and compares the impact of weekends and weekdays, especially considering the potential for ride-sharing. That is why this paper presents a further exploration of weekend travel behavior in MoD simulations.

## Method

**Figure 2.**
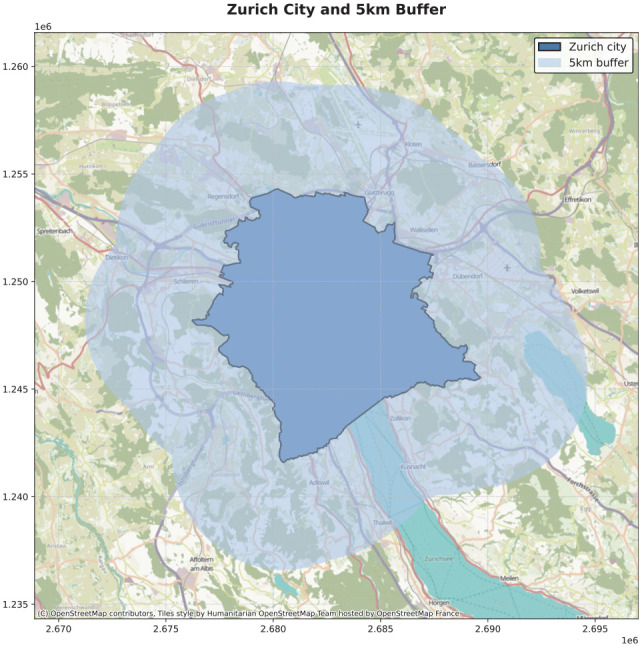
Analyzed study area.

This section describes the methods used in this study to develop an agent-based model for simulating an on-demand mobility service in Zurich, Switzerland, for the weekend (see Figure 2).

This study uses the multi-agent transport simulation framework MATSim (*28*) to develop the agent-based model. MATSim is a powerful simulation tool that can model detailed interactions between transport demand and supply. To represent the transportation system within MATSim, the following scenario data are required: a network representation consisting of links and nodes as well as the public transport infrastructure, travel demand data in the form of a synthetic population of agents with their corresponding travel plans, and additional transport elements such as facility locations and transit schedules.

The study area consists of the city of Zurich extended by a 5 km buffer covering an area of 383.56 km^2^ and contains a population of roughly 1.2 million. The transport network for the region is extensive, with 61,930 network links represented in MATSim and it facilitates various modes of transport. The 2015 Mikrozensus Household Travel Survey (HTS) by the Bundesamt für Statistik (BFS) provides insights into the travel behavior in the region. Within Zurich, 57% of the daily distance travelled by citizens of the Canton of Zurich is done by private car, 32% by public transport and 10% by foot, bike or e-bike with little to no presence of on-demand services such as Uber or Lyft. Given Zurich’s urban dynamics, MoD simulation is of particular relevance to the city as its transport planners contemplate sustainable transport solutions in the face of rapid urbanization. As a result, studies on the impact of MoD systems have and are currently being conducted in the city (*29*, *30*).

The travel demand model for the Zurich region for this study is extracted from a synthetic travel demand for Switzerland. The demand generation process for an average workday for Switzerland has been developed (*31*, *32*) and a calibrated simulation scenario for an average workday for the study region is presented in the paper (*33*). Below, the demand generation process for the weekend is described in detail.

### The Weekend Travel Demand Model

Several agent-based transport simulation studies conducted for Switzerland use the available Switzerland Baseline Scenario, a MATSim scenario created using an established synthetic population pipeline for Switzerland (*31*) based on the Eqasim framework (*34*, *35*). The Eqasim pipeline creates a realistic agent population matching the sociodemographics, mobility patterns, transport networks, and facilities of Switzerland. It draws on raw data, including census records, travel surveys, OpenStreetMap, and GTFS transit data. The resulting output of the pipeline is simulation files needed by MATSim, which include population, households, facilities, network, and transit vehicle and schedules files in XML format. The Switzerland Baseline scenario, based on the Swiss HTS, models an average working day and consists of a synthetic agent population that reproduces the sociodemographic characteristics and travel behavior of Switzerland. The HTS reports the daily travel behavior of nearly 60,000 respondents living in Switzerland and contains additional information that characterizes weekend travel. Over 22% of the trips recorded in the HTS are weekend trips, which provides an opportunity to capture weekend travel behavior.

Following an approach similar to that used to develop the average workday travel demand for Switzerland, an extension of the model has been developed in this study to create a weekend travel demand model for Switzerland. In Switzerland, Saturday and Sunday have distinctive trip patterns. As a result, the weekend model represents individual Saturday and Sunday synthetic travel demand. This extended model creates a synthetic population with households and persons and then defines daily activity patterns for the synthesized persons, using statistical matching approaches that use the weekend observations of the HTS to attach whole activity chains based on sociodemographic attributes. A generic pipeline for this synthesis process can be found in Hörl and Balac (*35*) and its application to Switzerland in Tchervenkov et al. (*31*) and Hörl (*32*). Below is a brief description of the weekend travel demand generation process while emphasizing the methodological differences to the weekday model.

#### The Synthesis Process

**Figure 3.**
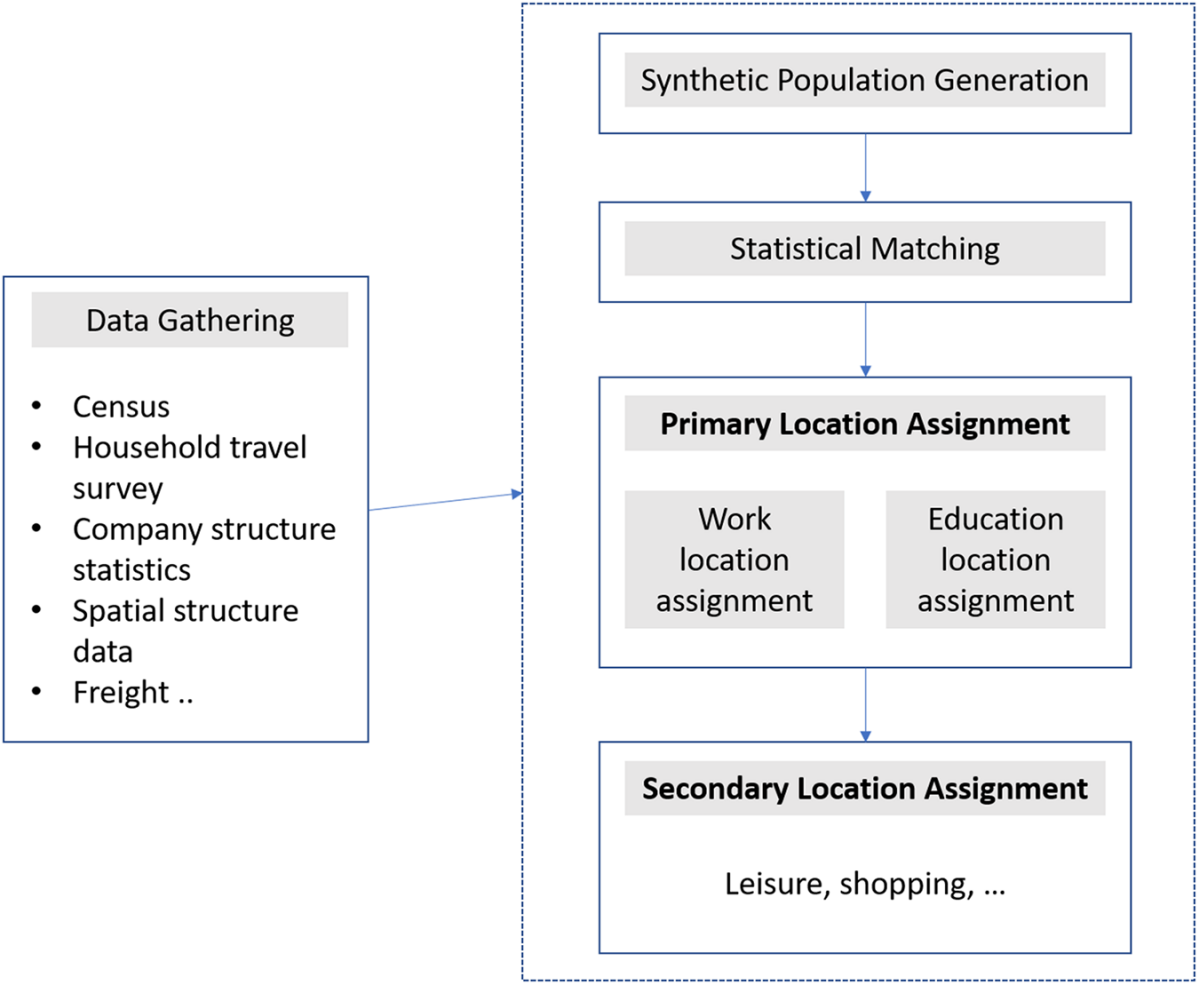
The synthesis process.

This section summarizes the synthesis process developed for the weekend travel demand model, highlighting key differences from the weekday model. The process is detailed sequentially: generation of synthetic population, statistical matching, assignment of primary and secondary activities, alongside unique considerations for the weekend model. Refer to Figure 3 for a pipeline diagram of the process. The synthesis process constitutes a chain of models to transform the data into a desired synthetic travel demand that can be used in an agent-based transport simulation.

**Synthetic Population Generation:** A synthetic population for Switzerland is generated from the census data. This involves using household-level and person-level data from the census to create agents representative of the population. The synthetic population generated for the different days of the week is the same, as the population is directly derived from the Swiss census. The activities are then imputed based on the distinct travel patterns of the different days of the week. The resulting synthetic population is then enriched with attributes such as driver’s license and car ownership derived from the Swiss HTS under the assumption of correlation between these attributes and activity chains. The enrichment process is detailed in the subsequent section.

**Statistical Matching:** This stage involves enriching each agent in the synthetic population with daily activity chains, which include start and end times, travel distances, and modes. The enrichment process uses a statistical matching algorithm that matches synthetic agents to observations in the HTS based on similar attributes (*35*, *36*). For the weekend model, only Saturday and Sunday activity chains are considered. The matching process is a multi-step process that involves defining a set of attributes at household and individual levels, noting down attribute vectors for both target and source observations, and then using a selection set level to sample source observations based on matching attributes and their weights, with the order of attributes relaxed when necessary to avoid overfitting. At the household level, five matching attributes—age, sex, type of residence municipality (urban, suburban or rural), marital status, and household size—are used. At the individual level, additional attributes, household income, number of cars, and number of bikes are considered. In the matching process, source observations are matched to the target based on these attributes and use their weights for sampling, while ensuring an adequate number of source observations to avoid overfitting. After matching, additional attributes are added to the synthetic persons, and activity chains are attached, detailing the purpose of activities and modes of transport.

**Figure 4.**
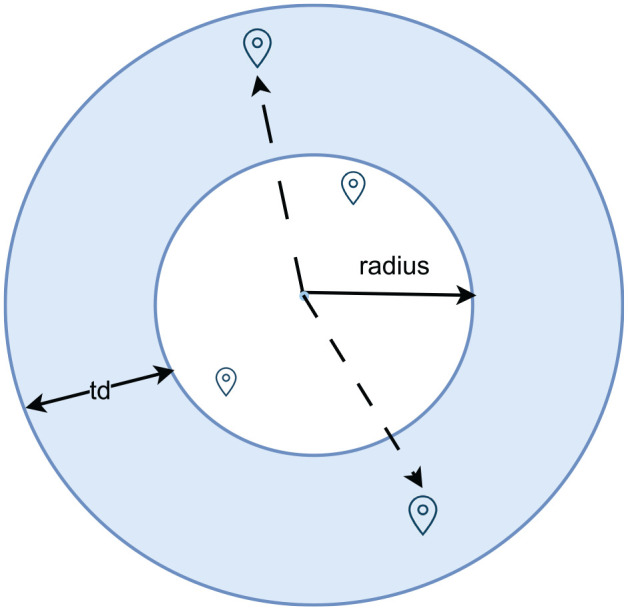
Location sampling in a donut-shaped region.

**Primary Activity Location Assignment:** This stage assigns locations for primary activities such as home, work, and education, basically activities expected to have a fixed location. In the weekend model, work and education activity locations are sampled based on a Swiss-defined facility type and function categorization (NOGA) with commute distances that are assigned using a probability distribution derived from HTS data. This approach contrasts with the weekday model, which assigns the nearest facility for commute trips based on origin–destination (OD) matrices from the Swiss structural survey data. To reduce selection bias, this stage also involves creating a selection region to choose facilities from, detailed in the donut-shaped location assignment seen in Figure 4. The donut-shaped location assignment model works by defining a selection region that excludes the immediate nearest facilities to prevent over-selection of facilities at the closest locations, while enforcing a probability-based selection from a certain range of distances. However, it does not impose a strict cutoff. Instead, the probability distribution used for trip distances is derived from empirical HTS data, which still includes short-distance trips. As a result, nearby locations may still be selected, but at a lower frequency, ensuring a representative distribution. Similarly, while the model discourages excessively long-distance commutes, it does not eliminate them entirely as longer trips may still occur based on the probability distribution. A unique consideration for the weekend model is the reduced number of education and work trips, which is reflected in the assignment process. All facilities designated as work locations in the NOGA were available to assign a workplace. The location assignment for work facilities on weekends did not distinguish between different types of work activities that might be more prevalent on those days owing to the data availability. Educational facilities active during weekends differ from those during weekdays, leading to additional categorization of educational facilities for the weekend model. Education facilities categories that are considered for the weekend include tertiary schools, driving schools, cultural education, IT training schools, language schools, sports and hobbies schools, adult training, and others.

**Secondary Activity Location Assignment:** Secondary activities include leisure, shopping, and other activities that require multiple places per agent. These activities can be performed in multiple places by each agent. Following a method outlined in the study (*37*), discrete locations are assigned to secondary activities while maintaining realistic distance distributions given travel times and modes in an activity chain. The output of the synthesis process is a travel demand scenario that can be used as an input to the MATSim simulation.

#### Validation of the Synthesis Process

**Figure 5.**
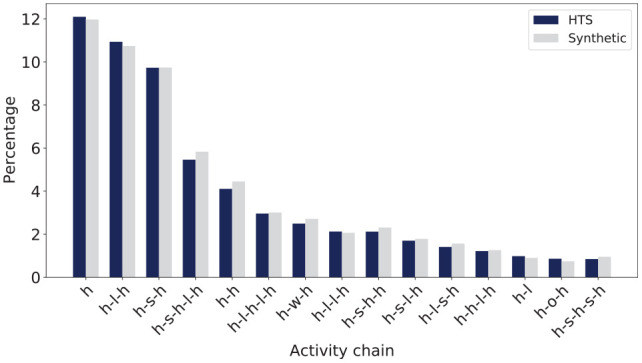
Activity chains for Saturday. *Note*: HTS = Household Travel Survey.

**Figure 6.**
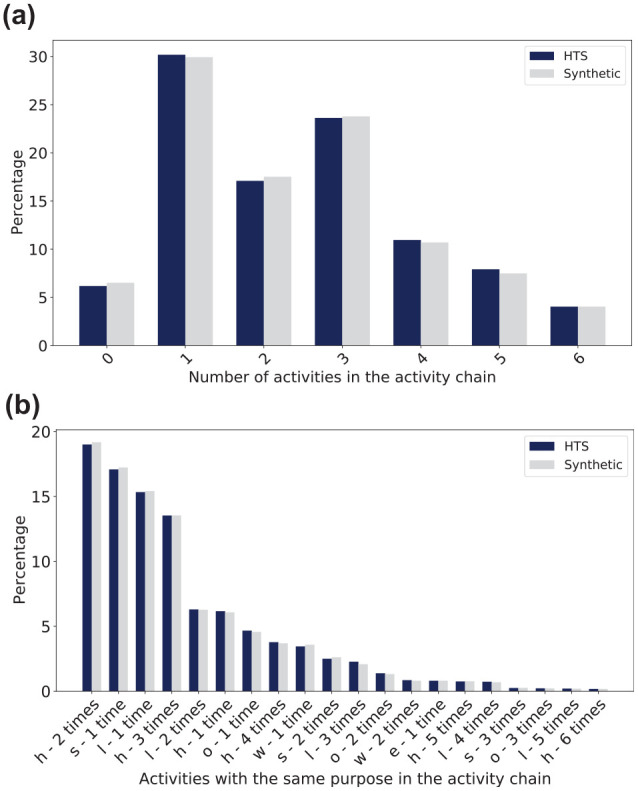
Number of activities for Saturday. (*a*) Activity counts. (*b*) Activity counts by purpose. *Note*: HTS = Household Travel Survey.

**Figure 7.**
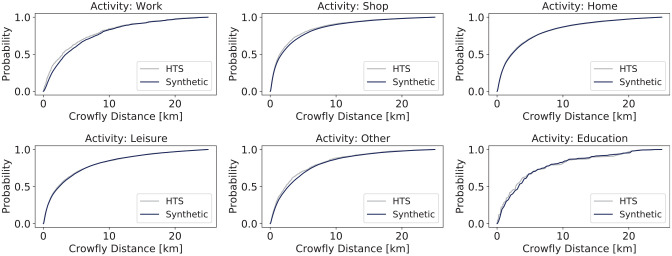
Cumulative distance distribution for Saturday.

It is important for the synthetic population that is generated from the synthesis process to match the overall patterns and capture the variability and distributions observed in real-world data. Figures 5 to 7 as well as the figures in Appendix B. show the validation process which demonstrates that the synthetic population generation for the weekend is reasonably successful in replicating the distribution of activity patterns and the travel distances for various purposes, and social demographic groups found in the HTS data.

First is an activity chain comparison that shows the distribution of various activity chains for the whole synthetic population. This is presented in Figure 5 for Saturday, and that of Sunday is shown in Figure B.2 in the Appendix. The figures compare the frequency of different activity chains, where each chain represents a sequence of activities, for example home–leisure–home (h-l-h) or home–shop–home (h-s-h). Figure 5 shows that the Saturday trips are mostly leisure trips, as one would expect, with home–leisure–home (h-l-h) and home–shopping–home (h-s-h) well represented in the synthetic population.

Figure 6*a* shows the activity counts, and Figure 6*b* shows the activity counts per purpose. The activity counts are done for activities that start from home. For a chain of h-l-h, this chain has only one activity, while a chain of h-s-h-l-h has three activities. When analyzing the number of activities in the activity chains, a count of zero activities represents individuals who stayed at home for the entire day or no report of activity. Overall, these figures show that the matching process produced reasonable results for the activity chains.

Figure 7 presents a cumulative distance distribution graph for the different activities, comparing the probability of travel distances between the synthetic population and HTS for different activities. The result shows that the location assignment process performed reasonably well as the synthetic model closely follows the HTS data, although there are a few deviations, particularly for education trips, which have very few observations in the travel survey.

The validation results suggest that further refinements could be made to improve the fidelity of the weekend synthetic population, particularly in better matching the HTS data for less common activity chains and distribution of activity counts. Still, the results are reasonable for this study and present an opportunity for further calibration to enhance the representativeness of the synthetic population.

After the synthetic population generation, a scenario-cutting process is used to extract the travel demand of the study region from the Switzerland model. This cutting process is described in detail in Hörl (*32*). The process filters agents based on time and space, considering interactions between the desired region to be cut out and the surrounding areas. When an agent enters or exits the region, a distinct “outside” activity is created. For example, if an agent’s activity chain is (home, leisure, home) but the leisure place is outside the study area, it would be recorded as (home, outside, outside, home) after the trips are adjusted. The mode of transportation between the two “outside” activities is labeled as an “outside mode” and is simulated in a way that ensures the correct activity timings are maintained.

#### Weekend Mode Choice Model

To study the transport mode choices that people make, a discrete mode choice (DMC) extension of MATSim (*34*, *38*) is used, which was already applied to study autonomous mobility on demand (aMoD) in Zurich (*33*).

A mode choice model for the average workday in Zurich already exists and is based on an empirical study conducted on mode choice patterns from a stated preference survey of the Zurich region that also considered emerging autonomous mobility (*33*). Authors in the study (*33*) formulated a multinomial discrete choice model with utility equations defined for car, public transit (PT), walk, bike, and aMoD and the mode choice variables including in-vehicle travel time, out-of-vehicle travel time (plus wait time and access/egress time), and travel cost. The utility formulation for aMoD is used for the MoD service in this study. The model formulation can be found in Appendix A.

For the weekend mode choice model estimation, the estimated average workday model is extended by calibrating the ASCs for each mode of transportation. This process aims to accurately represent the mode shares observed in the HTS during weekends. Applying this method here is based on the assumption that the VoTs and weekend travel dynamics align closely with the average workday. This assumption will be discussed later in this paper.

#### Calibration

The calibration process for the MATSim simulation was executed without a MoD service, as MoD trips are not present in the HTS data and thus cannot be validated. Adjustments were made to the ASCs for car, bike, and walk modes separately for Saturday and Sunday models. The ASC for PT remained at zero, as it served as the reference. These adjustments continued until we achieved a satisfactory alignment of modal shares and mode-specific distances with those recorded in the HTS. Relative error was used to evaluate the measure of deviation between observed and modeled trip shares.

**Figure 8.**
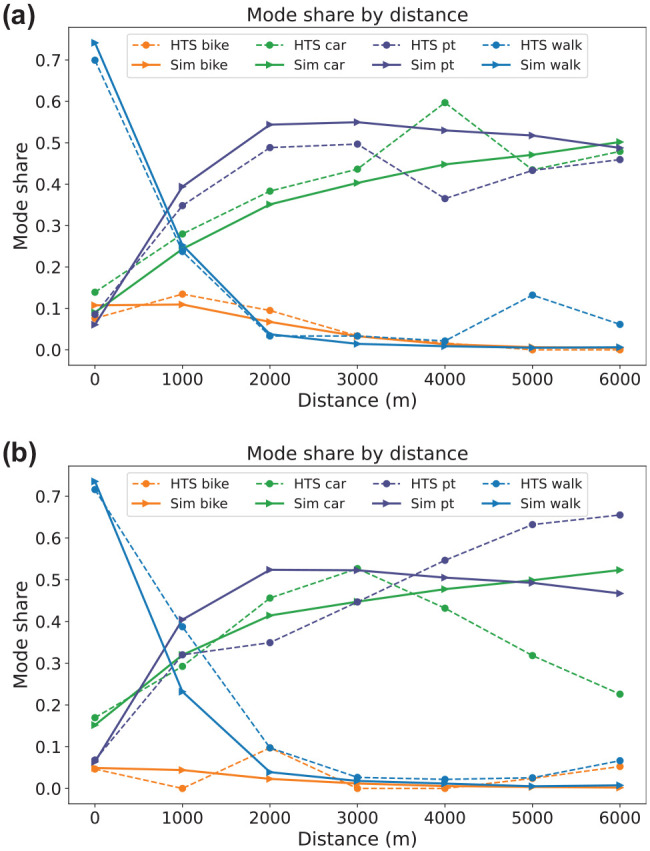
Mode share by distance from the calibration process. (*a*) Saturday. (*b*) Sunday.

The results of this calibration are detailed in Table 1 and Figure 8 which present a comparison between the mode shares of the HTS (serving as the reference data) and the calibrated scenario (Sim). The HTS and simulation data are filtered to include only the trips that occur entirely within the study region.

**Table 1.** Updated Parameters from Average Workday Model

	Avgday	Saturday	Sunday
$\beta_{\mathrm{ASC},\mathrm{car}}$	−0.8	−1.6108	−1.6632
$\beta_{\mathrm{ASC},\mathrm{bicycle}}$	0.1522	−0.640	−1.575
$\beta_{\mathrm{ASC},\mathrm{walk}}$	0.5903	−0.305	−0.6875

*Note*: HTS = Household Travel Survey.

The primary objective of the initial calibration was to achieve modal shares closely mirroring those of the HTS. Simultaneously, the second calibration objective was to ensure a reasonable distribution of distances per mode. Given the lack of reference data for trips exceeding 7 km, about 40 observations, we constrained our calibration by only comparing distances up to 7.5 km. This ensured that our simulation data adhered closely to the curve shape of the reference data. Figure 8 illustrates the modal shares of the calibrated modes, with distances segmented into 1 km bins. Figure 8 shows that the distance distributions for each mode depict the same trends as the HTS distributions. However, long-distance leisure walk trips are not well represented by the simulation.

### Weekend Simulation of MoD

In this study, the representation of an MoD service uses the MATSim demand-responsive transit (DRT) extension (*39*). The DRT extension was specifically developed to enable MATSim to simulate dynamic ride-sharing services, where vehicles can pick up and drop off passengers on request. A central dispatching system manages the fleet of vehicles and is responsible for scheduling and accepting incoming requests.

When a request is made, the dispatcher is presented with a list of available vehicles. The dispatcher algorithm traverses the list and assigns each request to the closest vehicle while ensuring that predefined constraints on wait and detour times for passengers are not violated. These constraints include: (1) ensuring that the overall travel time for passengers, including those currently in the vehicle or waiting for the vehicle, and the new customer, does not exceed predefined thresholds, and (2) ensuring that the expected boarding times for awaiting customers and the new customer fall within a requested time frame. If no suitable vehicle is available, the request is rejected. For further details on the DRT extension, refer to the study (*39*).

The MoD service offers a convenient door-to-door experience and allows for pooling, with a maximum seating capacity of four people. The service has a defined coverage area for only trips originating and ending within the study region. The service is not available for trips shorter than 250 m in Euclidean distance.

*MoD mode choice*: For the MoD service, the utility is calculated based on Equation 1. The equation includes x variables, which represent estimated trip-level attributes such as travel time and wait time. The β represents behavioral parameters that are estimated from empirical studies and quantify the share of the mode in the overall generalized costs for the trip. The ξ represents the elasticities of Euclidean distance on travel time ( $\xi_{\mathrm{TD}}$ ), cost ( $\xi_{\mathrm{CD}}$ ), and household income ( $\xi_{\mathrm{CI}}$ ). $x_{\mathrm{work}}$ and $x_{\mathrm{city}}$ are binary indicators that define trip purposes or locations, specifically for work-related and city-related trips. This model does not distinguish other activities such as shopping and leisure. It is important to note that the mode choice parameters for aMOD are not recalibrated owing to a lack of available data. As a result, we implicitly assume that the relative utility between public transport and MoD remains unchanged, while it may shift for other modes. The model parameters in Equation 1 are listed in Table A.1 in Appendix A.

(1) $$\begin{array}{c} {\tilde{v}}_{\mathrm{MoD}}\left(x\right)=\beta_{\mathrm{ASC},\mathrm{MoD}}\\ \;+\;\beta_{\mathrm{inVehicleTime},\mathrm{MoD}}\cdot \;\xi_{\mathrm{TD}}\cdot \;x_{\mathrm{inVehicleTime},\mathrm{MoD}}\;\\ \;+\;\beta_{\mathrm{accessEgressTime},\mathrm{MoD}}\;\cdot \;x_{\mathrm{accessEgressTime},\mathrm{MoD}}\\ \;+\;\beta_{\mathrm{waitingTime},\mathrm{MoD}}\;\cdot \;x_{\mathrm{waitingTime},\mathrm{MoD}}\\ \;+\;\beta_{\mathrm{work},\mathrm{MoD}}\;\cdot \;x_{\mathrm{work}}\\ \;+\;\beta_{\mathrm{highAge},\mathrm{MoD}}\;\cdot \;\left[a_{\mathrm{age}}\;\geq \;60\right]\\ \;+\;\beta_{\mathrm{cost}}\;\cdot \;\xi_{\mathrm{CD}}\;\cdot \;\xi_{\mathrm{CI}}\;\cdot \;x_{\mathrm{cost},\mathrm{MoD}} \end{array}$$

Travel times and costs are provided by the MATSim network router, which is estimated dynamically during the simulation’s evolution depending on traffic, the number of vehicles, pricing, departure time, and so on. Wait times and additional delays for the MoD service observed throughout the day are fed back into the choice model based on the estimation of the average wait times per defined zones and time intervals. This is to add additional behavioral realism as travelers make decisions; they should be informed by the operator of the expected wait time or delays in their area and during the time of day that they would make the trip so as to decide to use the service. Therefore, during every iteration of the mobility simulation, wait times and travel time information of agents who use the MoD service are tracked for each time interval and zone. Then the mean is calculated over all waiting times and delays experienced in the zone. When no waiting is observed in a zone and time interval in a particular iteration, the existing value is maintained, and in the case when none exists, a default wait time of 10 min is applied. A square grid size of 1 km is used with a time interval of 15 min. These values were selected from conducting a sensitivity analysis for various grid sizes and time intervals. The results of these analyses are described in detail in Kagho (*40*).

While the MATSim DRT extension used for simulating the MoD service allows for rejection constraints to be set, such as rejecting passengers when a fixed maximum wait time and/or maximum detour time (the time a passenger is willing to tolerate while the vehicle picks up another passenger) is exceeded, these constraints are not considered in this study as rejection would not be captured during the mode choice. Instead, the on-demand mobility service is penalized through the feedback of wait time and delay time metrics, detailed above. Additionally, since a door-to-door DRT scheme is applied in this study, there is no access or egress time, and the parameter is set to zero. The cost is taken from the study by Hörl et al. (*33*) where 0.6 CHF/person km is determined to cover the cost of operating a 4,000 fleet for Zurich city. This is reasonable to use across the different fleet sizes as estimated in the study (*41*), that the cost of taxi services would be about 0.41 CHF/person km for the canton of Zurich.

### Simulation Scenarios

This study focuses on the potential impact of considering weekend travel demand when modeling MoD services. Focusing on the weekend demand aims to address a gap in the current research landscape and explore an important aspect of operational planning for on-demand services.

A baseline scenario which represents the current travel demand state without the MoD service is defined for each of the modeled days: an average workday, Saturday and Sunday. The transport network encompasses a comprehensive representation of the study area, including its road infrastructure and transit lines. Different transit schedules have been generated for the weekend, using 18 and 19 January 2020 for Saturday and Sunday, respectively, and 15 January 2020 (a Wednesday) to represent a typical workday.

Eight MoD service scenarios are defined for each of the simulation days. Each MoD scenario is differentiated by simulating different fleet sizes ranging from 3,000 to 10,000 in 1,000 intervals. See Table 2.

**Table 2.** Simulation Scenarios

Scenario	Simulation day	MoD fleet size range
Baseline	Average workday, Saturday, Sunday	N/A (No MoD)
MoD Scenario 1–8	Average workday, Saturday, Sunday	3,000–10,000

*Note*: MoD = mobility on demand.

The study focuses on the following areas:

- Operational planning policies: The impact of various fleet sizes on the MoD level of service and vehicle use is examined.
- Service reliability and availability: How the demand varies temporarily and spatially between the days, which can affect service reliability, is examined.
- Modal shift implications: The implications for transport policies on modal shifts are drawn out by examining how the shifts occur for the different days modeled, and potentially revealing why.

## Results

The following section presents the results for the scenarios simulated, providing insights into the impact of weekend travel demand on service reliability, operational planning policies, and policy implications.

### Operational Planning Policies

**Figure 9.**
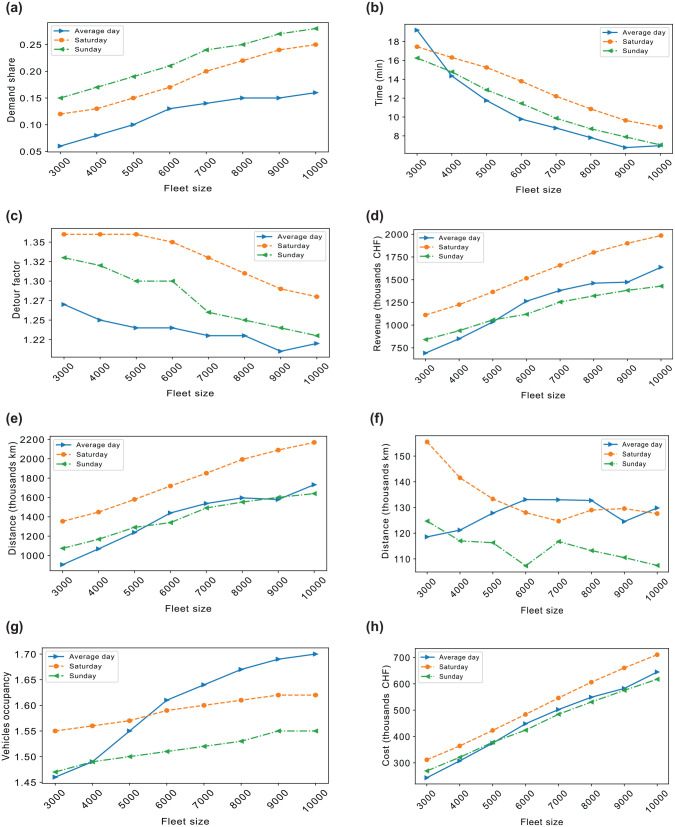
Service level metrics and vehicle operational performance by fleet size: (*a*) Mobility on demand (MoD) share. (*b*) Average wait time. (*c*) Average distance detour factor. (*d*) Total revenue. (*e*) Total vehicle driven distance. (*f*) Total empty vehicle distance. (*g*) Average vehicle occupancy. (*h*) Total operational cost.

The operational planning policies of MoD services, particularly fleet sizing, are examined here with regard to the weekend travel demand. Figure 9*a* to *d* shows the influence of varying fleet sizes on the level of service metrics, which reveals potential impacts on the MoD’s operational efficiency and effectiveness during weekends.

Since the fare structure is fixed at a rate of 0.6 CHF/km, regardless of the fleet size, one can examine how the fleet size influences the demand patterns across different days, allowing for insights into how potential users may weigh service wait times and delays against the utility of alternative modes of transport as well as the resulting revenue. It can be observed that the demand patterns are different depending on the day. Thus, the number of vehicles required to provide reliable service varies with the fluctuating demand. By considering these different demand patterns, we can better address the key question of the optimal fleet size. For example, Saturday and Sunday, which have a higher share of demand for the MoD service, do not necessarily have a larger difference in wait times than the average day; however, the travelers may experience longer delays during the weekends, possibly owing to the spatial distribution of the demand and is reflected in the longer detours compared with the average work day. Furthermore, distances travelled on Saturday are longer, bringing in more revenue that could cover operational costs.

Similarly, how various fleet sizes perform in vehicle operational performance areas such as travel distance, operational cost, empty distance traveled, and vehicle occupancy are also highlighted in the last four subfigures in Figure 9*e* to *h*. The cost of maintaining a fleet of vehicles, $C_{\mathrm{fleet}}$ , is calculated using the cost calculator suggested in the study (*41*) and adapted in Hörl et al. (*33*) where the authors feed the cost model in the study (*41*) with measured values from an agent-based transport simulation, as opposed to using “best-guess” predictions for fleet utilization and empty distances.

(2) $$\begin{array}{c} C_{\mathrm{fleet}}=c_{\mathrm{perDistance}}.d_{\mathrm{fleetDistance}}+\\ c_{\mathrm{perTrip}}.n_{\mathrm{numberOfTrips}}+\\ c_{\mathrm{perVehicle}}.n_{\mathrm{fleetSize}} \end{array}$$

where $d_{\mathrm{fleetDistance}}$ describes the total fleet distance including both the vehicle empty kilometers driven and service kilometers, $n_{\mathrm{numberOfTrips}}$ describes the total number of rides given, and $n_{\mathrm{fleetSize}}$ describes the number of vehicles in the fleet. The following cost units are derived from the study (*41*) where $c_{\mathrm{perDistance}}$ is the average cost per vehicle kilometer, $c_{\mathrm{perTrip}}$ is the average cost per trip, $c_{\mathrm{perVehicle}}$ is the average total costs of operating a vehicle per day.

$$\begin{array}{c} c_{\mathrm{perDistance}}=0.098\mathrm{CHF}/\mathrm{km}\\ c_{\mathrm{perTrip}}=0.375\mathrm{CHF}\\ c_{\mathrm{perVehicle}}=33.30\mathrm{CHF}\left(\mathrm{per}\mathrm{day}\right) \end{array}$$

From an operator’s standpoint, tracking distances, costs, and vehicle occupancy are essential to ensure optimal fleet performance at a minimal cost. For instance, although the average day and Sunday demonstrate similar trip distances compared with Saturday, the “empty” distances vary, likely owing to differing demand densities. This observation is supported by the vehicle occupancy results, which hint at increased ride pooling for Saturday and very low pooling for Sunday. Notably, this study does not account for group travel, implying that vehicle occupancy could be underestimated for weekend trips, which have a higher potential for group travel.

Operational costs play a critical role in determining fleet size. While having enough vehicles to minimize wait times and delays is essential, maintaining the fleet’s costs is equally important. The results show that the costs between the average day and weekend are not very different. Saturday only incurs, on average, about a 10% increase in cost compared with Sunday and an average day. However, the increased revenue generated over the weekend offsets this higher expenditure.

If using empty vehicle distance and vehicle occupancy as a determinant of a reasonable fleet size that can serve the different days, one can see that if only the average day is considered, the size of the fleet that would be selected would be different than when the weekend is considered. Furthermore, Table 3 illustrates the operational performance across different days for a fleet size of 5,000 which is used in subsequent analysis. By examining the empty distance ratio and net income, the cost of operating this fleet can be covered by the revenue generated on all the days, with an average wait time of 15 min across all days, even though the detour time factors are quite high on average owing to congested routes, the median values range from 2 to 2.5, meaning that travelers are still willing to choose the MoD service even when their travel time is increased by this factor. The distance detour and time detour factors are calculated as the ratio between the actual distance or time traveled in a shared ride and the direct travel distance/travel time between the origin and destination.

**Table 3.** Operational Performance for the Scenario with a Fleet Size of 5,000

Metric	Average day	Saturday	Sunday
Demand	231,166	2,71,291	226,842
Avg waiting time [min]	11.75	15.25	12.87
Avg distance detour factor	1.24	1.36	1.3
Avg time detour factor	2.98	4.04	3.69
Person km [1,000 pkm]	1,724.59	2,275.68	1,762.4
Total vehicle driven distance [1,000 km]	1,238.5	1,579.7	1,291.04
Total vehicle occupied distance [1,000 km]	1,110.67	1,446.39	1,174.73
Total empty vehicle distance [1,000 km]	127.83	133.32	116.31
Empty ratio	0.1	0.08	0.09
Rejections	0	0	0
Rejections rate	0	0	0
Avg travel time [min]	20.57	17.1	15.56
Cost [1,000 CHF]	374.56	423.05	378.09
Revenue [1,000 CHF]	1,034.75	1,365.41	1,057.44
Net income [1,000 CHF]	660.19	942.36	679.35

### Service Reliability and Availability

**Figure 10.**
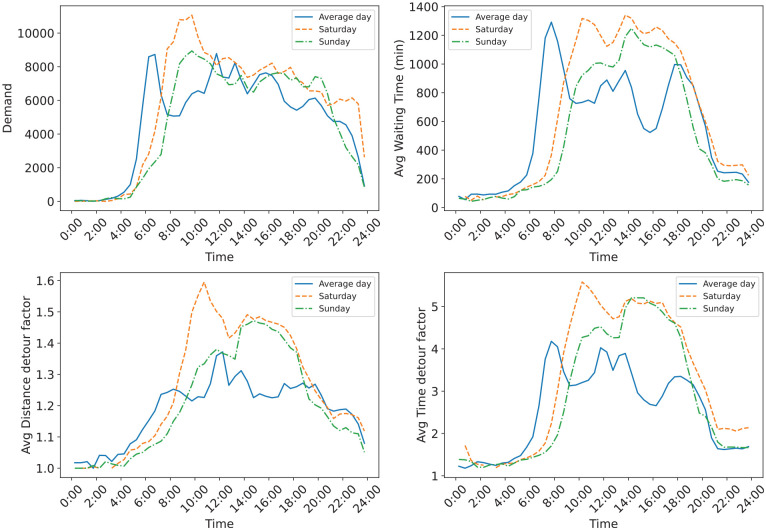
Temporal distribution of travel patterns.

This section presents the findings related to the service reliability and availability of the MoD service, specifically focusing on the impact of weekend travel demand. The analysis looks at how reliable the service is over the different days of the week, both temporally and spatially, by identifying demand peaks. This helps to examine how the fluctuating demand during the weekends might affect service reliability in comparison with the weekdays and then be able to identify the specific time slots or regions that experience higher demand and may require additional attention. As expected, from Figure 10, the average day has distinct peaks following the typical morning and afternoon commute patterns in Switzerland, compared with the weekend, although the evening peak is missing. However, for the weekend, one can observe that trips start a bit later, and the demand is almost flat most of the day, especially on Sunday. What is also noticeable is that wait times and delays are higher toward the evening period, which could be attributable to overall congestion of the roads caused by car users.

**Figure 11.**
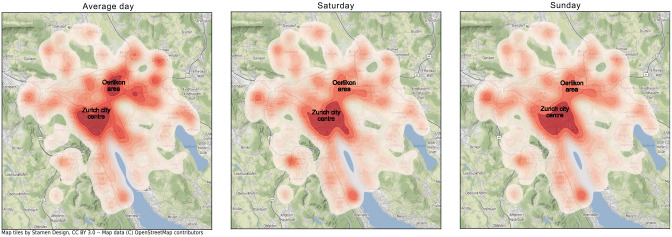
Spatial distribution of on-demand mobility trips.

Similarly, the spatial distribution of demand differs between weekdays and weekends, as can be seen from Figure 11. One can observe two clusters of originating MoD trips for the average day (Zurich city center and Oerlikon), compared with weekends, where trips are concentrated in the center. This makes sense as Oerlikon is a commercial center which would attract commuting trips that use the MoD. Considering these differences in temporal and spatial patterns, attention needs to be paid to the outcomes of dispatching and rebalancing methods to account for these differences, among other factors. Even though rebalancing was not implemented in this study, it remains an important factor to consider when evaluating MoD system performance.

### Modal Shift Analysis

**Figure 12.**
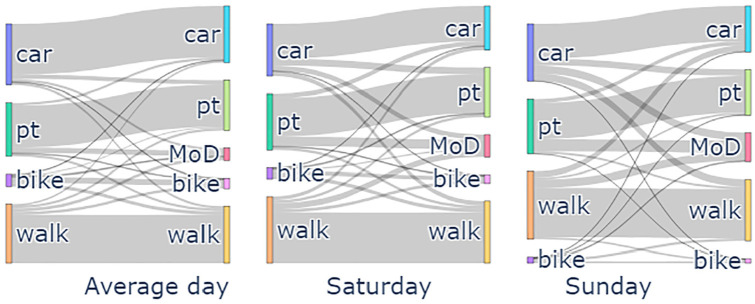
Modal shift dynamics between the baseline scenario and mobility on demand (MoD) scenario.

**Figure 13.**
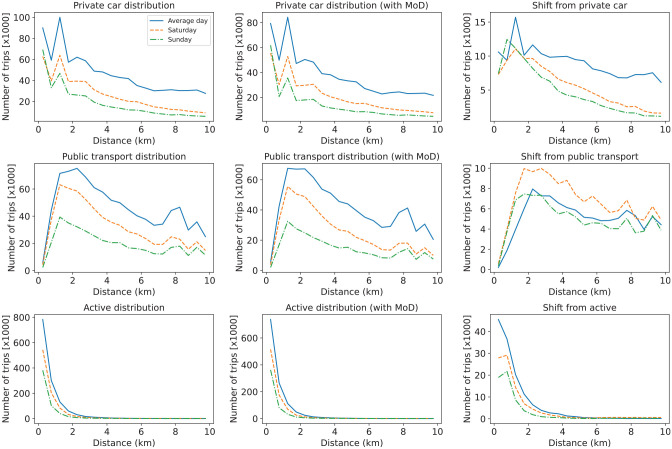
Distance distribution of trips by modal shifts. *Note*: MoD = mobility on demand.

Here, a modal shift analysis helps to examine how weekend travel demand affects a shift toward using MoD services, considering whether individuals were more inclined to switch from private vehicles or other travel modes to MoD services during weekends. To do this, MoD trips from the 5,000 vehicles MoD scenario for each day of the week are identified in the baseline scenarios where there was no MoD service. The set of trips is compared, and the results of this analysis are shown in Figures 12 and 13. The percentage values of the shifts between modes visualized in the Sankey diagram are presented in Table C.1 in Appendix C. First, from the Sankey diagram presented in Figure 12 one can see how much the private car mode is shifting to the MoD service compared with PT and active modes. On the other hand, one can observe that more PT trips are being replaced during the weekend. The reason for this can be observed in Figure 13, which shows that travelers shift from PT to the MoD service mostly for long-distance trips during the weekend, compared with the shift from private cars. This suggests that policy design for attracting travelers to shift from private cars to shared mobility would have to take into consideration the different days of the week, especially in ensuring that there is less shift from PT for weekend trips, perhaps as an example, developing intermodal policies for PT and MoD services during the weekends.

## Discussion and Conclusion

The study’s hypothesis, centered around the relevance of including weekend travel data for effective MoD operational planning and policy decisions, is supported by the evidence produced in the study. The role of weekend travel demand in MoD service efficiency was examined through various key areas: operational planning policies, service reliability and availability, and policy and planning implications.

The results presented the nuances of weekend travel demand and its impact on MoD simulations. Variations in demand patterns across the days of the week were shown to affect the choice of the optimal fleet size required to deliver reliable service. Unsurprisingly, the weekend, particularly Saturday, showed higher demand levels for MoD services. It was observed that the increase in demand during weekends did not dramatically alter wait times compared with weekdays. However, there were longer delays, potentially attributed to the differing spatial distribution of demand. The importance of considering such variations when deciding on fleet size becomes apparent, as the optimal fleet size would be different if only weekdays were considered. Still, future research needs to explore the optimal fleet composition that accounts for both weekday and weekend varying demand patterns in a whole week simulation.

The cost analysis showed that the fleet’s operational cost does not vary much between the average day and the weekend. Although Saturday showed a slight increase in cost, the corresponding increase in revenue more than compensated for this, making it potentially worthwhile choosing a larger fleet expenditure to accommodate weekend demand.

Analyzing the service reliability and availability revealed distinct patterns in the temporal and spatial distribution of the demand between weekdays and weekends. While weekdays saw peak demand times aligning with typical commute hours, weekend demand started later and remained consistent throughout the day. Also, two major clusters were identified for weekdays (Zurich city center and Oerlikon), while weekend trips were more centralized. These patterns provide valuable input for refining dispatching and rebalancing methods in the MoD operational strategy. Furthermore, a modal shift to MoD service was observed, particularly over the weekends, with shifts from private car, PT and active modes, especially for long-distance PT trips.

In conclusion, including weekend travel demand in MoD simulations is essential for transport planning. By considering weekend travel demand, MoD services can be optimized to meet varying patterns of demand effectively, resulting in enhanced operational efficiency and service reliability. Furthermore, weekend travel demand data can inform policy decisions about encouraging a shift away from private vehicle use toward more sustainable modes.

While informative, the results of this study should be interpreted with a degree of caution. Certain limitations are worth noting. A major limitation of the study is estimating the weekend mode choice model, whereby an existing average workday model has been extended by calibrating the ASCs of the different modes to represent mode shares observed in the HTS for the weekends. Using the same VoTs for the weekdays and weekends supposes that the behavioral preferences that determine the mode of transport on weekdays remain constant and extend to the weekends. Also, there is an implicit assumption that the relative utility between public transport and MoD remains unchanged, while it may shift for other modes. Therefore, it is important to note that these assumptions may oversimplify the complexities of travel behavior (*42*, *43*). As a result, using weekday VoTs to calibrate weekend models could introduce bias and potential inaccuracies into the model. However, using average weekday VoTs is practical without specific weekend survey data. Therefore, future research in this area should address this limitation identified in this study. Collecting specific weekend travel data to calibrate weekend models more accurately would be beneficial. Activity-specific parameters can be estimated by capturing the different activity profiles on the weekend. Alternatively, a mixed-mode choice model could be explored, which allows for heterogeneity in VoT across individuals and potentially captures the variances between weekday and weekend travel behaviors, as well as accounting for trip purposes.

Furthermore, for weekends, leisure and shopping activities often play a major role in travel behavior, however, in developing the weekend travel demand model for this study, work and education activity locations are assigned as the primary location for the population. This was done for practicality and consistency with the existing weekday model. Implementing this method is based on the assumption that even work and education activities on the weekend happen at fixed and consistent locations, just as during the weekdays. It also allows for using the existing infrastructure and provides a baseline for comparison with weekday travel behavior. Future research should explore the impact of not prioritizing work and education activities as primary locations on weekends. This would involve a more technical extension of the current model, requiring detailed activity pattern data and potentially new algorithms for activity assignment.

This study broadly compares how the differences in travel demand patterns between weekends and weekdays can affect the policies drawn from MoD simulations for the city of Zurich. While developed methodology can be applied to other regions, whether the results can be generalized and transferred to other regions is unknown. This is left for future work to show.

## Supplemental Material

sj-pdf-1-trr-10.1177_03611981251346454 – Supplemental material for Mobility On Demand: What About the Weekend? Supplemental material, sj-pdf-1-trr-10.1177_03611981251346454 for Mobility On Demand: What About the Weekend? by Grace O. Kagho, Milos Balac and Kay W. Axhausen in Transportation Research Record
