# Supplementary material for: Mobility On Demand: What About the Weekend?
Source: Transp Res Rec. 2025 Aug 15;2679(11):68–84. doi: 10.1177/03611981251346454 (PMC13046270; doi:10.1177/03611981251346454)
Supplement: sj-pdf-1-trr-10.1177_03611981251346454 – Supplemental material for Mobility On Demand: What About the Weekend? [file sj-pdf-1-trr-10.1177_03611981251346454.pdf]

APPENDIX

A.Mode choice model Formulation for Zurich region

The mode choice model estimation for the weekend is based on the average workday model developed from an empirical study conducted on mode choice patterns from a stated preference survey of the Zurich region that also considered emerging autonomous mobility (**?**). **?** formulated a multinomial discrete choice model with utility equations defined for car, PT, walk, bike, and aMoD.

The model equations are presented in Equations 1, 4, 5, and 6 and the corresponding parameter values are presented in Table A.1. Within the model, the utility U_i is computed for each mode (i), while choice variables are represented as x. The marginal utility parameters are denoted as β, and the alternative specific constants (ASCs) are denoted as $\beta_{\text{ASC,i}}$. The mode choice variables encompass factors such as in-vehicle travel time, out-of-vehicle travel time (including wait time and access/egress time), and travel cost. The $\beta_{\text{ASC,car}}$ had to be adjusted from 0.223 shown in Table A.1 to -0.8 to achieve a good model fit. This is explained in detail in **?**.

The utility for *car* is defined by the equation

$$\begin{aligned} u_{car} = &\beta_{\text{ASC,car}} \\ &+ \beta_{\text{inVehicleTime,car}} \cdot \xi_{\text{TD}} \cdot x_{\text{inVehicleTime,car}} \\ &+ \beta_{\text{work,car}} \cdot x_{\text{work}} + \beta_{\text{city,car}} \cdot x_{\text{city}} \\ &+ \beta_{\text{cost}} \cdot \xi_{\text{CD}} \cdot \xi_{\text{CI}} \cdot x_{\text{cost,car}} \end{aligned} \tag{1}$$

The attribute x_{work} defines whether the trip originates or ends at a *work* activity, and the attribute x_{city} describes whether the trip starts or ends inside of the city area of Zurich. ξ_{TD}, ξ_{CD} and ξ_{CI} are elasticities of Euclidean distance on travel time and on cost and elasticity of household income on cost and they are defined in the utility equations where λ describes additional model parameters that need to be estimated.

$$\xi_{TD} = \left(\frac{x_{\text{euclideanDistance}}}{\theta_{\text{referenceDistance}}}\right)^{\lambda_{TD}}, \quad \text{and} \quad \xi_{CD} = \left(\frac{x_{\text{euclideanDistance}}}{\theta_{\text{referenceDistance}}}\right)^{\lambda_{CD}}. \tag{2}$$

$$\xi_{CI} = \left(\frac{a_{\text{householdIncome}}}{\theta_{\text{referenceIncome}}}\right)^{\lambda_{\text{CI}}} \tag{3}$$

The utility for *PT*:

$$\begin{aligned} u_{pt} = &\beta_{\text{ASC,pt}} \\ &+ \beta_{\text{inVehicleTime,train}} \cdot \xi_{\text{TD}} \cdot x_{\text{inVehicleTime,train}} \\ &+ \beta_{\text{inVehicleTime,other}} \cdot \xi_{\text{TD}} \cdot x_{\text{inVehicleTime,other}} \\ &+ \beta_{\text{inVehicleTime,feeder}} \cdot x_{\text{inVehicleTime,feeder}} \\ &+ \beta_{\text{waitingTime,pt}} \cdot x_{\text{waitingTime,pt}} \\ &+ \beta_{\text{accessEgressTime,pt}} \cdot x_{\text{accessEgressTime,pt}} \\ &+ \beta_{\text{headway,pt}} \cdot x_{\text{headway,pt}} \\ &+ \sum_{G} \beta_{\text{pt quality,G}} \cdot x_{\text{pt quality,G}} \\ &+ \beta_{\text{cost}} \cdot \xi_{\text{CD}} \cdot \xi_{\text{CI}} \cdot x_{\text{cost,pt}} \end{aligned} \tag{4}$$

$x_{\text{inVehicleTime,train}}$ is the time a traveler spends in a train. Whereby there are additional feeder modes such as buses or trams to a train, $x_{\text{inVehicleTime,feeder}}$ is considered as the travel time in these feeder modes, while $x_{\text{inVehicleTime,other}}$ is zero. In the absence of a rail leg on the chosen route, travel time in busses, trams or ferries is considered as $x_{\text{inVehicleTime,other}}$ while $x_{\text{inVehicleTime,feeder}}$ is set to zero.

The attribute $x_{\text{ptQuality}}$ quantifies the accessibility to public transport at any place in Switzerland as defined by the Federal Office of Land Use in Switzerland, based on proximity to PT stops and stations and the frequency of the respective lines. It is defined on five levels $G \in \{\text{A}, \text{B}, \text{C}, \text{D}, \text{None}\}$ with A as the highest.

The utility for cycling:

$$\begin{aligned} u_{bicycle} = &\beta_{\text{ASC,bicycle}} \\ &+ \beta_{\text{travelTime,bicycle}} \cdot \xi_{TD} \cdot x_{\text{travelTime,bicycle}} \\ &+ \beta_{\text{highAge,bicycle}} \cdot [a_{\text{age}} \geq 60] \end{aligned} \tag{5}$$

where a represents agent-level attributes, in this case, the age of each agent.

The utility for walking is defined as:

$$\begin{aligned} u_{walk} = &\beta_{ASC,walk} \\ &+ \beta_{travelTime,walk} \cdot \xi_{TD} \cdot x_{\text{travelTime,walk}} \end{aligned} \tag{6}$$

There was a need to correct for an increased attractiveness of the walk mode; therefore, Equation 6 was adjusted by including an additional penalty term. For shorter travel time, the penalty tends to zero while for travel time equal to the threshold of 120 minutes, there is a large offset of -100 as shown in Equation 7

$$\begin{aligned} u_{walk_i} = &u_{walk} - \\ &\exp\left(\log 10 \cdot \frac{x_{\text{travelTime, walk}}}{\theta_{\text{walkThreshold}}}\right) + 1 \end{aligned} \tag{7}$$

	Parameter	Estimate	
Car	$\beta_{\text{ASC,car}}$	$0.224*$	
	$\beta_{\text{inVehicleTime,car}}$	-0.019	$[\text{min}^{-1}]$
	$\beta_{\text{work,car}}$	-1.161	
	$\beta_{\text{city,car}}$	-0.459	
Public Transport	$\beta_{\text{ASC,pt}}$	0.0	
	$\beta_{\text{inVehicleTime, feeder}}$	-0.045	$[\text{min}^{-1}]$
	$\beta_{\text{inVehicleTime, other}}$	-0.012	$[\text{min}^{-1}]$
	$\beta_{\text{inVehicleTime, train}}$	-0.007	$[\text{min}^{-1}]$
	$\beta_{\text{transferTime, pt}}$	-0.012	$[\text{min}^{-1}]$
	$\beta_{\text{accessEgressTime, pt}}$	-0.014	$[\text{min}^{-1}]$
	$\beta_{\text{headway, pt}}$	-0.030	$[\text{min}^{-1}]$
	$\beta_{\text{pt quality, B}}$	-1.744	$[\text{min}^{-1}]$
	$\beta_{\text{pt quality, C}}$	-1.641	$[\text{min}^{-1}]$
	$\beta_{\text{pt quality, D}}$	-0.965	$[\text{min}^{-1}]$
	$\beta_{\text{pt quality, None}}$	-1.089	$[\text{min}^{-1}]$
Bike	$\beta_{\text{ASC,bicycle}}$	0.152	
	$\beta_{\text{travelTime,bicycle}}$	-0.126	$[\text{min}^{-1}]$
	$\beta_{\text{highAge,bicycle}}$	-2.659	$[a]$
Walking	$\beta_{\text{ASC,walk}}$	0.590	
	$\beta_{\text{travelTime,walk}}$	-0.046	$[\text{min}^{-1}]$
AMOD	$\beta_{\text{ASC,AMoD}}$	-0.061	
	$\beta_{\text{inVehicleTime,AMoD}}$	-0.015	$[\text{min}^{-1}]$
	$\beta_{\text{waitingTime,AMoD}}$	-0.093	$[\text{min}^{-1}]$
	$\beta_{\text{work,AMoD}}$	-1.938	
	$\beta_{\text{highAge,AMoD}}$	-2.6588	$[a]$
Other Parameters	β_{cost}	-0.089	$[\text{CHF}^{-1}]$
	λ_{CI}	-0.817	
	λ_{CD}	-0.221	
	λ_{TD}	0.115	
	$\theta_{\text{referenceDistance}}$	39	[km]
	$\theta_{\text{referenceIncome}}$	12.260	[CHF]

Table A.1. Parameters of the discrete mode choice model

B. Validation of the synthesis process for Weekend Synthetic Population

The validation of the synthetic population generation process for Sunday is shown in the following figures, which consist of the activity chains, activity counts and distance distribution comparisons between the household travel survey data and the generated synthetic travel demand for Saturday and Sunday. The process outline in the Methodology appears to capture the overall trends in activity patterns and travel distances.

Activity Chains Characteristics

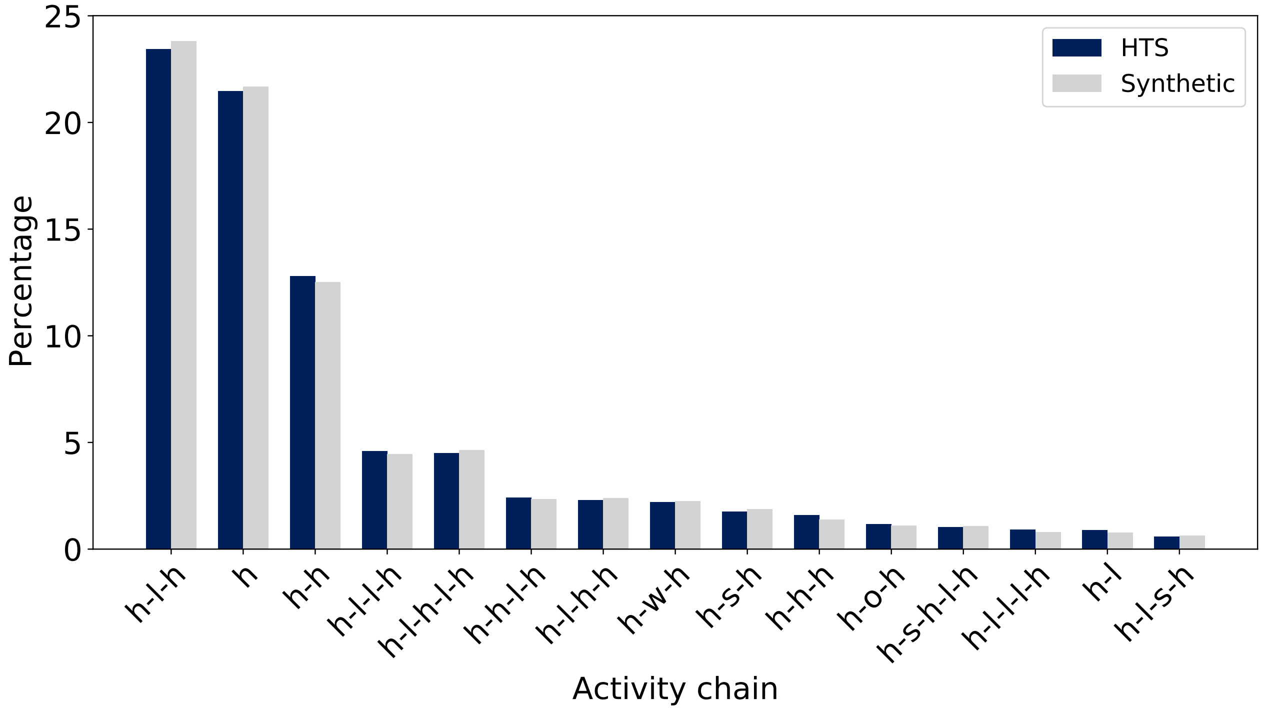

Figure B.1. Activity chains for Sunday

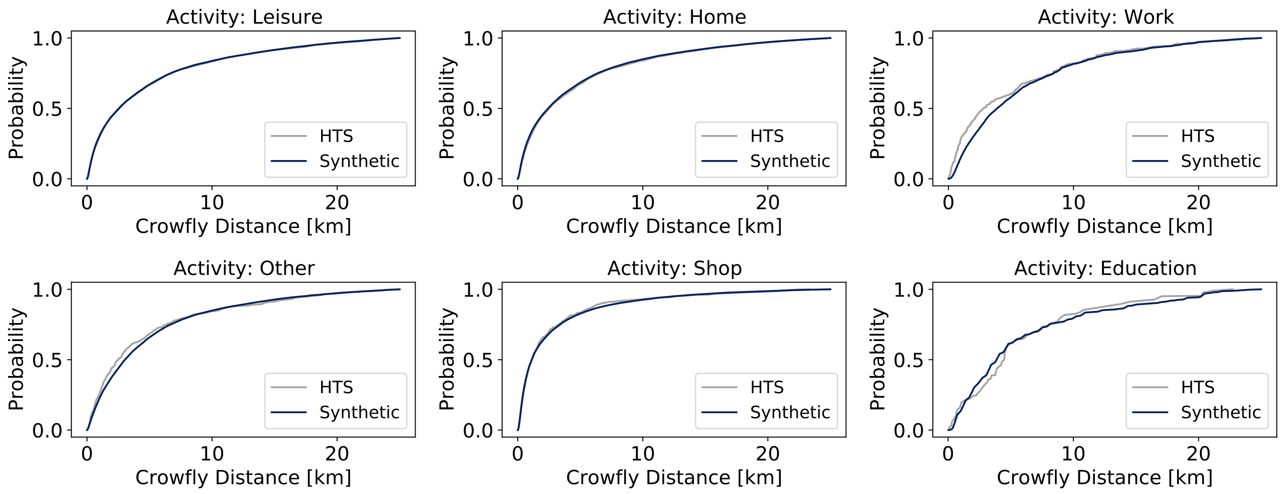

Figure B.2. Cumulative distance distribution for Sunday

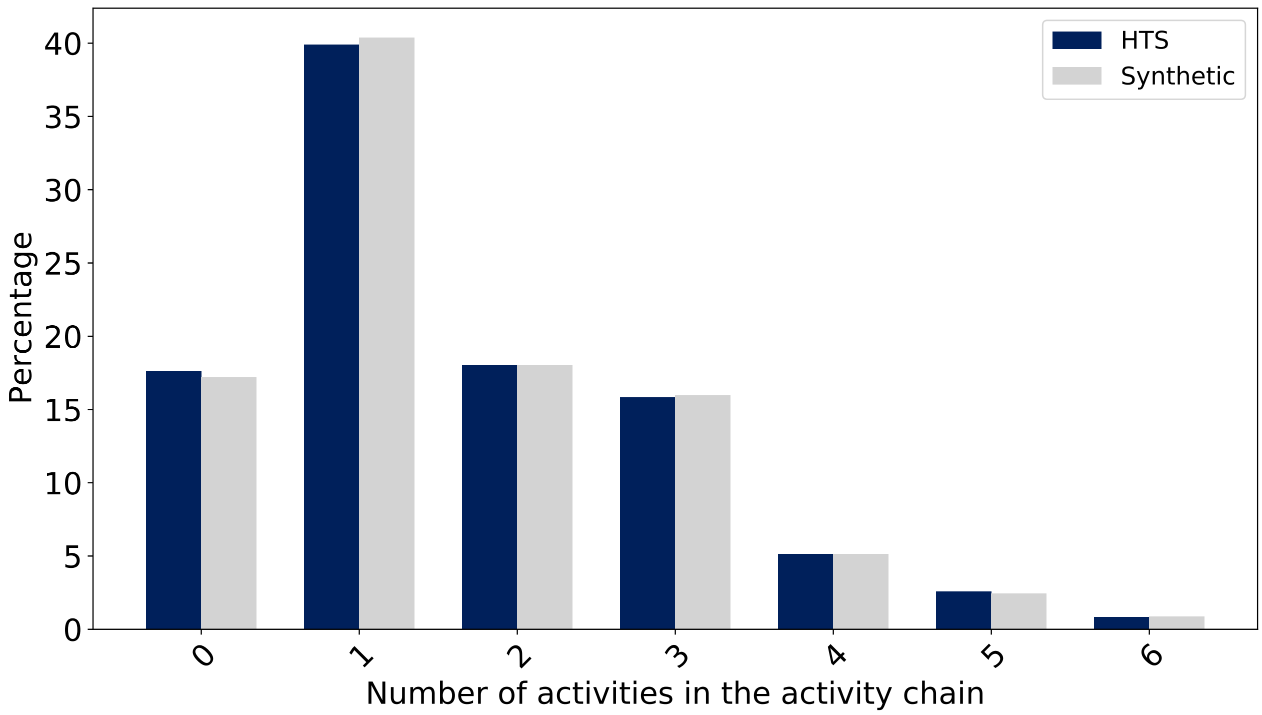

(a) Activity counts

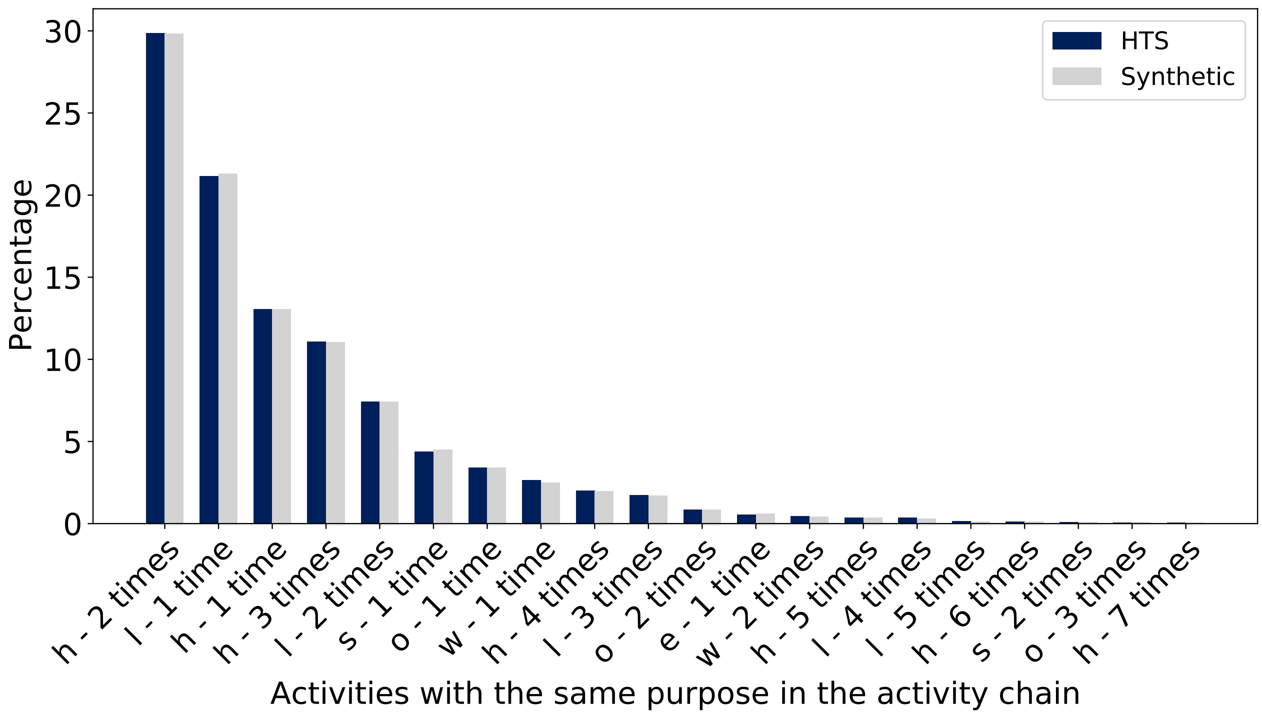

(b) Activity counts by purpose

Figure B.3. Number of activities for Sunday

C. Modal shift analysis

A modal shift analysis was carried out to examine whether individuals are more inclined to switch from private vehicles or other travel modes to MoD services during weekends. To conduct this analysis, MoD trips from a scenario involving 5000 MoD vehicles for each day of the week were identified and compared against the baseline scenario where no MoD service was available. This comparison allows for an understanding of the extent to which travelers shift from private cars, public transit, and active modes (such as walking and biking) to MoD services during weekends. The results are shown in Table C.1.

Table C.1. MoD Modal shift analysis

From/To	Car	PT	MoD	Bike	Walk
Average day					
Car	82.9	7.1	4.4	1.2	4.4
PT	5.1	78.4	10.1	1.5	4.8
Bike	5.8	9.7	8.2	47.8	28.5
Walk	3.4	4.4	5.7	5.0	81.5
Saturday					
Car	66.96	13.06	9.74	1.36	8.9
PT	7.94	68.02	17.26	1.32	5.45
Bike	6.28	10.1	12.52	38.37	32.73
Walk	5.54	4.53	9.38	4.5	76.08
Sunday					
Car	60.93	11.6	13.68	0.94	12.86
PT	7.12	66.45	22.17	0.37	3.87
Bike	9.42	5.71	16.19	28.98	39.7
Walk	9.09	3.05	11.61	2.94	73.11
